# *Nano**sculpt*: A methodology for generating complex realistic configurations for atomistic simulations

**DOI:** 10.1016/j.mex.2016.03.002

**Published:** 2016-03-10

**Authors:** A. Prakash, M. Hummel, S. Schmauder, E. Bitzek

**Affiliations:** a Department of Materials Science and Engineering, Institute I, Friedrich-Alexander-Universität Erlangen-Nürnberg (FAU), 91058 Erlangen, Germany; b Institut für Materialprüfung, Werkstoffkunde und Festigkeitslehre, University of Stuttgart, Pfaffenwaldring 32, 70569 Stuttgart, Germany

**Keywords:** Experimentally informed atomistic simulations, Molecular dynamics simulations, 3D materials science, Atom probe tomography, Digitized microstructure, Ni-base superalloy

## Abstract

Atomistic simulations have now become commonplace in the study of the deformation and failure of materials. Increase in computing power in recent years has made large-scale simulations with billions, or even trillions, of atoms a possibility. Most simulations to-date, however, are still performed with quasi-2D geometries or rather simplistic 3D setups. Although controlled studies on such well-defined structures are often required to obtain quantitative information from atomistic simulations, for qualitative studies focusing on e.g. the identification of mechanisms, researchers would greatly benefit from a methodology that helps realize more realistic configurations. The ideal scenario would be a one-on-one reconstruction of experimentally observed structures. To this end, we propose a new method and software tool called *nano**sculpt* with the following features:

• The method allows for easy sample generation for atomistic simulations from any arbitrarily shaped 3D enclosed volume.
• The tool can be used to build atomistic samples from artificial geometries, including CAD geometries and structures obtained from simulation methods other than atomistic simulations.
• The tool enables the generation of experimentally informed atomistic samples, by e.g. digitization of micrographs or usage of tomography data.

## Graphical abstract

**Figure:**
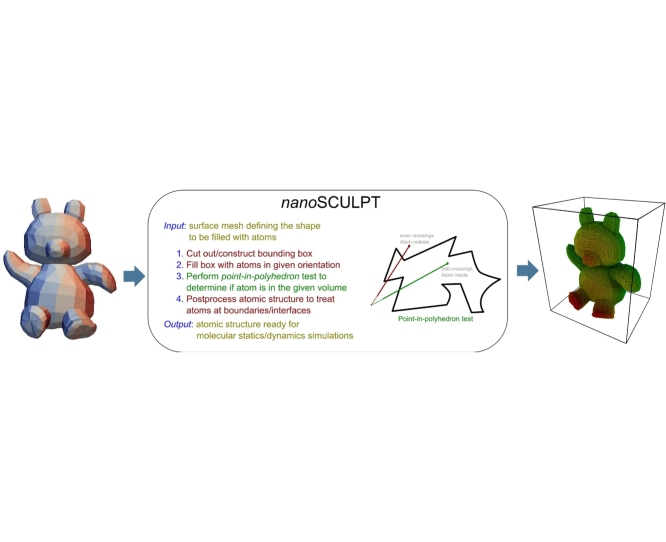

## Method details

### Background

Large-scale atomistic simulations have now established themselves as a valuable tool in advancing our understanding of the mechanical properties, deformation behavior, and failure of materials. Such simulations have led to unprecedented insights into fundamental deformation mechanisms in metallic materials. Examples include the study of dislocation and grain boundary mediated processes in nanocrystalline metals, investigation of precipitate strengthening, simulation of fracture processes, study of dislocation nucleation controlled plasticity in nano-objects and deformation behavior of nanocontacts.

The determination of quantitative information like obstacle strength or the dislocation drag coefficient requires well-controlled, simplified atomistic simulation setups, which often involve quasi-2D geometries with periodic boundary conditions (PBC) or perfectly planar interfaces. However, the use of overly simplified setups has been shown to artificially suppress important deformation mechanisms. A recent study on curved precipitates in Ni-base superalloys, for instance, revealed interaction mechanisms between matrix dislocations and the misfit dislocation network, which were not observed in prior simulations on planar interfaces [1]. Other examples include the suppression of jogs, kinks or crack front curvature effects in quasi-2D simulations of fracture processes and the suppression of dislocation nucleation at surface steps by using perfectly planar surfaces in the study of nanowires.

Parallel to the increased use of atomistic simulations in materials science, advances in experimental methods like atom probe tomography (APT) [2], automated serial sectioning techniques [3] and 3D X-ray tomographic microscopy [4], which provide high resolution 3D information about the microstructure, have given rise to the emerging field of three-dimensional materials science [5]. Although the 3D information provided by these methods is increasingly used in continuum scale simulation methods like crystal plasticity full-field simulations, the use of experimentally-informed atomistic simulation setups has remained scarce. A primary reason is the lack of resolution of many of these methods, which limits their use in constructing atomistic samples.

A further reason for this near absence of experimentally informed atomistic simulations is simply the lack of a method and a widely available program that can be used to directly construct atomistic samples from 3D data sets. This also explains why mesoscale simulation techniques like phase field models, level-set methods or interface tracking methods are not yet routinely used to provide input structures for atomistic simulations. Most available programs are currently limited to the generation of simple shapes like cylinders, spheres, octahedron and/or other polyhedra [6], [7], [8] (see also [9]). The fundamental idea here is to generate a large enough sample, that is generally cuboidal, and “*sculpt*” out the required shape. Although relatively complex shapes can be generated using e.g. series of Boolean operations, the process is, nevertheless, restricted to perfectly geometric shapes and smooth surfaces.

Here we present a methodology and software tool to create *atomistic samples from arbitrarily shaped 3D datasets*, including experimentally obtained data, structures generated from mesoscale simulation methods, and engineering components. This software tool opens up the possibility to make use of readily available real or synthetic microstructures, e.g. from the 3D Materials Atlas Project [10] or generated by software tools like DREAM3D [11]. With the available methods to generate arbitrary dislocation arrangements [12] and to impose elastic displacement fields [13], e.g., of cracks, together with robust analysis and visualization tools, the study of defect–defect interactions is now possible in more realistic microstructures.

### Methodology of structure generation using *nano**sculpt*

Although the working principle of *nano**sculpt* can be easily generalized to non-crystalline structures like glasses, for simplicity, crystalline structures will be used in the following description and examples. The methodology of *nano**sculpt* can be summarized by the following steps:

(1) Identify the shape of individual crystallites that is to be filled with atoms.
(2) Cut out and/or add additional surfaces to conform to the desired simulation box.
(3) Obtain crystallographic information on the individual crystallites under consideration.
(4) Fill the identified volume with atoms according to the crystallographic information obtained in *Step 3*.
(5) Postprocess the atomistic structure to treat atoms at boundaries and/or interfaces.

**Fig. 1:**
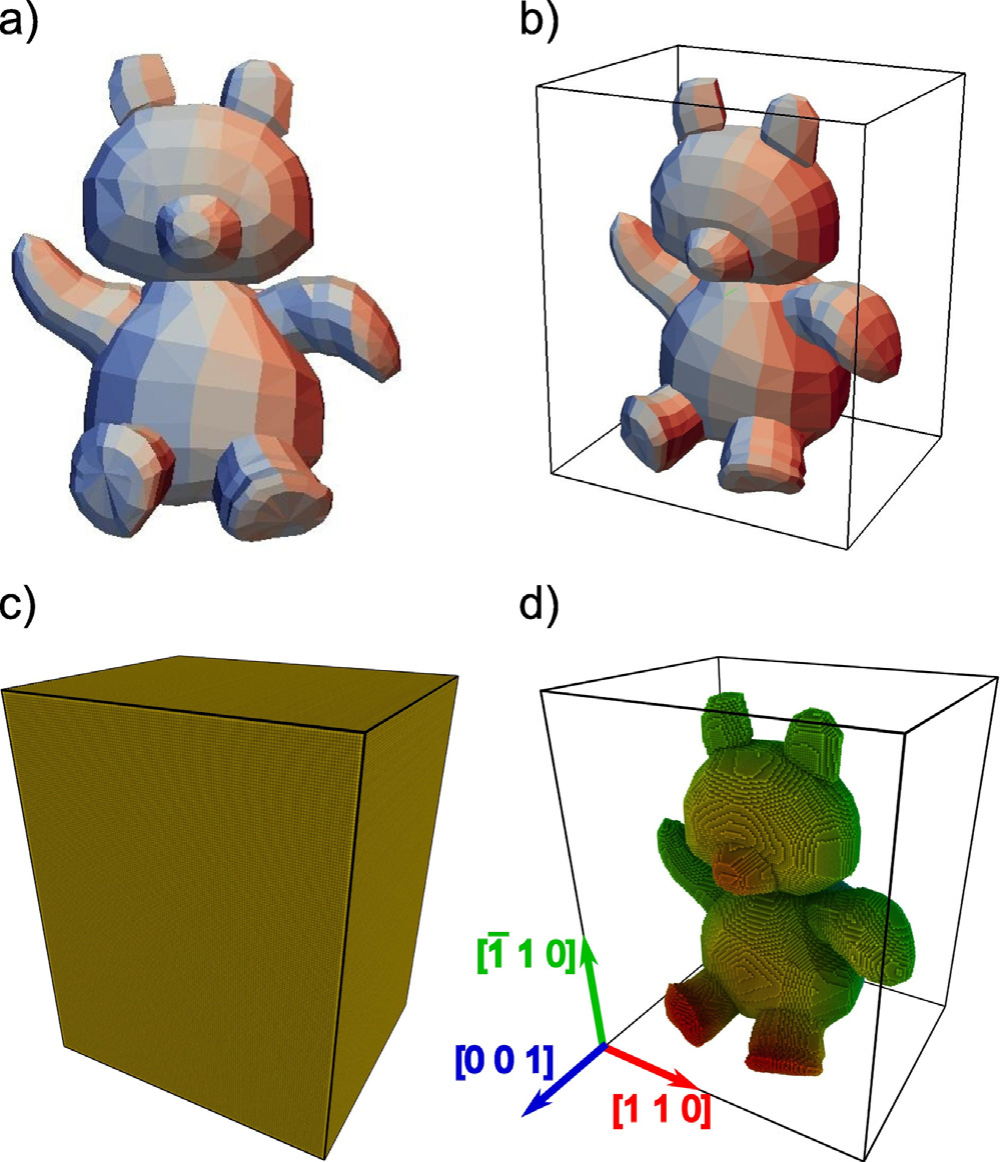
Demonstration of the methodology of *nano**sculpt* using a Teddy bear: (a) in the first step, we identify the volume (here, teddy bear) that is to be filled with atoms and define a surface mesh to delimit this volume; (b) we now define a bounding box using the geometric limits of the teddy bear; (c) the bounding box is then completely filled with atoms using appropriate crystallographic information—here, fcc structure, lattice constant of 0.408 nm (Au) and orientation [110]|| *x*, [001]|| *z* axes; (d) extraction of atoms inside the defined volume of the teddy bear using a point-in-polyhedron test. In a post-processing step, the so created atomistic sample can then be treated for grain boundaries and interfaces; atoms are colored according to the element (Au) in (c) and depth in (d).

The individual steps of the sample generation process are illustrated with the help of a teddy bear in Fig. 1, and are described below in detail.

#### (1) Identify the shape of individual crystallites that is to be filled with atoms

The first step in atomistic sample generation is to clearly identify the region of interest for further simulations and to define a surface mesh to enclose this volume. The desired sample can be obtained by any of the following techniques:

(i) Artificial geometries obtained by computer aided drafting/drawing (CAD), including sketching, as shown in the teddy bear example above.
(ii) Simulated microstructures obtained e.g. using phase-field models, level-set methods or interface tracking methods.
(iii) Digitization of images obtained by transmission electron microscopy (TEM) or scanning electron microscopy (SEM), possibly in combination with serial sectioning.
(iv) Three dimensional tomography, e.g. atom probe, electron or X-ray tomography.

The surface mesh is essentially a set of points, together with facet information that provides the connectivity between points. An accurate description of this enclosing surface is critical since it has a significant effect on the surface topology of the atomistic structure, particularly for small (nanoscale) structures. Depending on the technique used, additional intermediate stages may be required to obtain a well defined surface mesh that completely encloses the identified region of interest. For instance, in the case of digitization of image stacks, isosurfaces defined by greyscale or color can be used to demarcate different crystalline regions in the sample (see *Digitization of experimental micrographs* section). In the case of tomography data obtained by atom probe measurements, iso-density surfaces of specific chemical species can be used to define the interface between different phases [1].

#### (2) Cut out and/or add additional surfaces to conform to the desired simulation box

In certain cases, the surface mesh obtained in the previous step may not be directly usable for atomistic simulations and may require additional processing. This may be necessitated e.g., if the loading conditions require planar surfaces or cuboidal simulation boxes. Such additional surfaces can be generated by performing Boolean operations on the surface mesh obtained in *Step 1* and cutting out the desired shape. Further details are provided in the application examples presented in *Method validation* section.

#### (3) Obtain crystallographic information on the individual crystallites under consideration

In order to fill the enclosed volume with atoms, information on the orientation, structure and lattice parameter of individual crystallites is required. If the orientation information is not directly determined from the experimental method used to detect the shape of individual crystallites in *Step 1*, complementary experiments, like e.g. correlative TEM, electron backscatter diffraction (EBSD) or X-ray diffraction, are required. The lattice parameter is usually defined by the interatomic potential that is to be used in further simulations.

#### (4) Fill the identified volume with atoms using the crystallographic information obtained in Step 3

Once the crystallographic data has been established, we proceed to fill the enclosed volume (determined in *Step* 1) that defines the shape of the crystallite. This is done in 3 stages: In a first stage, we set up a bounding box around the enclosed volume under consideration. The size of this bounding box is usually obtained from the geometric limits of the volume to be filled. In a second stage, the bounding box is completely filled with atoms in the defined crystallographic orientation. This is done by rotating the primitive lattice vectors of the desired lattice structure into the given orientation and then placing atoms at all integer multiples of these lattice vectors, starting from a chosen origin. For amorphous structures, the atomic positions within the bounding box have to be determined by other simulation techniques, e.g., using the melt-quench approach or reverse Monte Carlo simulation [14]. In the third stage, we perform a *point-in-polyhedron* test to evaluate if each atom lies inside the pre-determined volume or not, and retain only those atoms in the region of interest.

For the point-in-polyhedron test, we leverage algorithms of computational geometry, like e.g. the *ray-casting* algorithm or the *winding number* algorithm. In *nano**sculpt* in particular, we make use of the ray-casting algorithm and derive our implementation from that of [15]. Briefly, the ray-casting algorithm works as follows: for every point (atom) under consideration, we test the number of times a ray, starting from the atom and going in any arbitrary direction, crosses the enclosed volume in question. If the number of ray crossings is odd, the point is inside the volume. If the number of crossings is even, the point is outside.

It must be pointed out that for two phase structures, i.e. for cases where the crystallite to be filled is deemed to be an inclusion in a matrix, the bounding box can be much larger (see examples on the *γ*/*γ*′ microstructures below) and is filled with atoms using crystallographic data of one of the phases (inclusion or matrix). The subsequent point-in-polyhedron test then retains atoms either inside (inclusion) or outside (matrix) the crystallite volume.

#### (5) Postprocess the atomistic structure to treat atoms at boundaries and/or interfaces

In the presence of interfaces—generated either by assembly of individual crystallites, or due to a single crystallite embedded in a matrix—in the final atomistic structure, there is a high likelihood of atoms being very close to each other. To avoid this, atoms at the interface are removed in an iterative manner following the approach described in [16], if the interatomic distance is less than a given critical value, which is usually taken to be between 60% and 80% of the nearest neighbor distance as defined by the potential under consideration.

## Method validation

We present various examples that illustrate the generation of atomistic samples using *nano**sculpt*, from the different techniques mentioned above in *Step 1*. All atomistic samples in the examples below have been visualized with Ovito [17].

### Sample structures obtained from artificial geometries

Using artificial geometries is perhaps the easiest and most exploited method to obtain realistic starting structures for atomistic simulations. For demonstrative purposes, we present two examples.

**Fig. 2:**
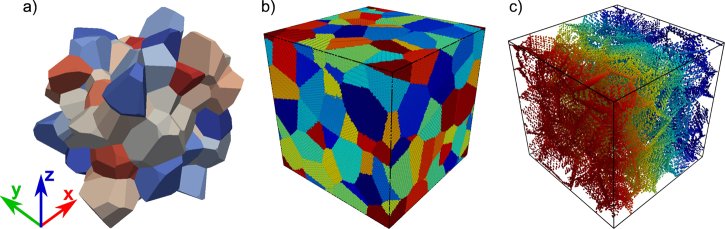
Generation of an atomistic sample via Voronoi tessellation: (a) geometric structure defining the surface topology of each grain; (b) atomistic structure for Al (*a*_0_ = 0.405 nm) with a simulation box of 42.7 nm × 42.7 nm × 42.7 nm obtained by using *nano**sculpt*; (c) structure showing discarded atoms—only those atoms with neighbors within the critical distance of 0.185 nm are removed from the atomistic sample in (b). The color map in (a) and (b) is chosen so as to identify individual grains. For clarity, the atoms are colored by their *x*-coordinate in (c).

Voronoi tessellation has become the method of choice in studies involving the deformation behavior of polycrystalline, in particular nanocrystalline, materials. It is an elegant method to generate artificial microstructures that mimic real polycrystals. In comparison to geometries generated with mono-dispersive models that use cubes, hexagon shaped 2D microstructures or equisized polyhedra, Voronoi tessellation results in microstructures that are seemingly more similar to those observed in experiments. In principle, Voronoi tessellated microstructures can be constructed by choosing “*seeds*” or grain centers and dividing the simulation box into cells such that any point contained inside a cell is at a distance that is lowest to its own grain center than to all other centers in the box. Consequently, atomistic configurations of such structures can be generated by periodically replicating a lattice structure, in a given orientation, around a grain center. When the distance of an atom to the current grain center is no longer smaller than its distance to any of the other grain centers, we stop with the structure generation for the current grain. An alternative approach is to use algorithms like Qhull [18], since each cell is formed by a convex hull enclosing all points within the cell. This is also the method of choice in *nano**sculpt*. Fig. 2 shows an artificial microstructure generated by means of the Voronoi tessellation. It contains 100 grains with approximately 4.6 million atoms. The average grain size of the microstructure is 11.5 nm.

**Fig. 3:**
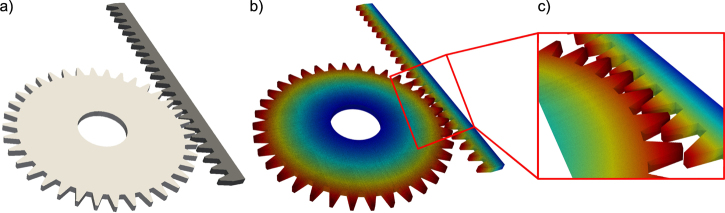
Generation of an atomistic structures from CAD geometries—presented here is an example of a rack and pinion system; (a) surface topology as obtained by CAD. The gearwheel has a diameter of 780 nm and the height of a gear tooth is 54 nm. The linear rack has a length of 1.4 μm with a height of 107 nm. The thickness of both components is 30 nm; (b) atomistic sample generated for Si (*a*_0_ = 0.543 nm). The structure contains approximately 570 million atoms in the gearwheel and 130 million atoms in the linear rack. Atoms are colored by their distance to the center in the gearwheel, and by their distance to the base in the linear rack.

A second method of obtaining artificial structures is to use CAD tools to generate the surface geometry. As an example, we present the CAD model of a rack and pinion system (Fig. 3(a)). Such systems find their typical applications in nano/micro electro-mechanical systems (NEMS/MEMS) and devices. The gearwheel has a diameter of approximately 780 nm with a tooth height of 54 nm. The linear rack has a length of 1.4 μm and a height of 107 nm. The thickness of both components is 30 nm. The surface mesh of the gearwheel contains 288 vertices and 572 facets, while that of the rack contains 180 vertices and 272 facets. The atomistic structure, with silicon as the material, comprises approximately 570 million atoms in the gearwheel and 130 million atoms in the linear rack.

### Sample structures obtained from other simulation methods

Artificial geometries like the ones discussed above often result in structures which display properties that are far removed from those of realistic microstructures. For instance, in the case of the Voronoi tessellated microstructures discussed above, only planar interfaces are formed. Additionally, the distribution of statistically relevant topological entities, like e.g. number of triple junctions, grain boundary area, number of neighbors per grain, do not conform to those observed in experiments.

An alternative approach for obtaining artificial structures is to make use of simulation tools like grain-growth models or phase-field models. This has the added advantage that the obtained structures are based on strong physical principles. As a result, the topological and structural features are closer to those observed in experiments. Indeed, these methods can be used to optimize microstructures so as to mimic statistical distributions obtained from experiments.

**Fig. 4:**
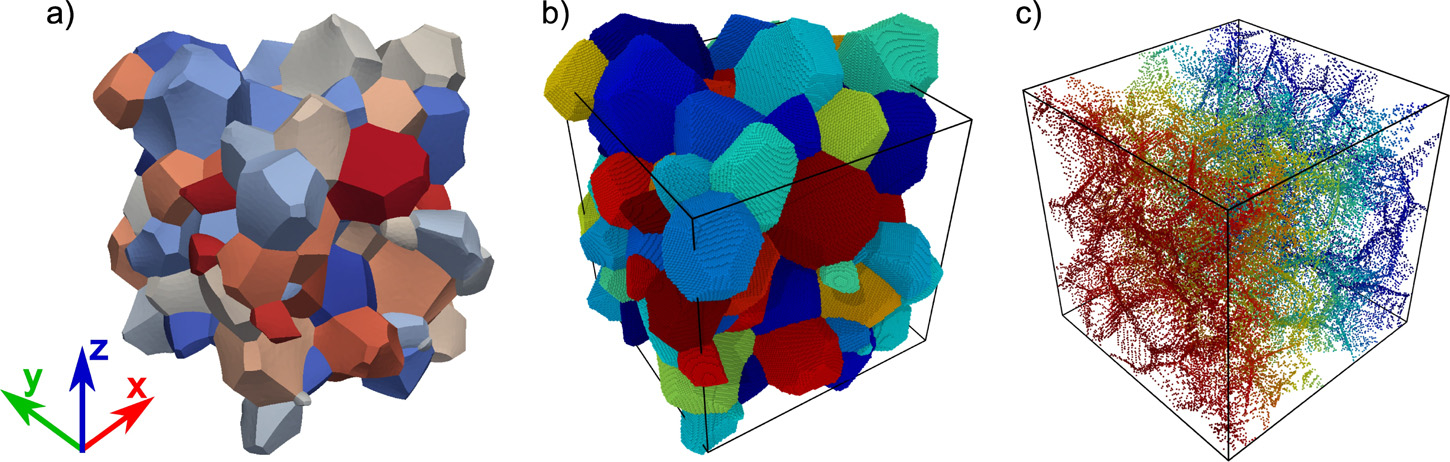
Generation of an atomistic sample using a snapshot structure of a grain growth simulation: (a) geometric structure defining the surface topology of each grain. Note the presence of curved GBs, in comparison to the completely planar GBs observed in the Voronoi tessellation (Fig. 2(a)); (b) atomistic structure for Al (*a*_0_ = 0.405 nm) with a simulation box of 42.7 nm × 42.7 nm × 42.7 nm obtained by using *nano**sculpt*; (c) structure showing discarded atoms—only those atoms with neighbors within the critical distance of 0.185 nm are removed from the atomistic sample in (b). The color map in (a) and (b) is chosen so as to identify individual grains. For clarity, the atoms are colored by their *x*-coordinate in (c).

**Fig. 5:**
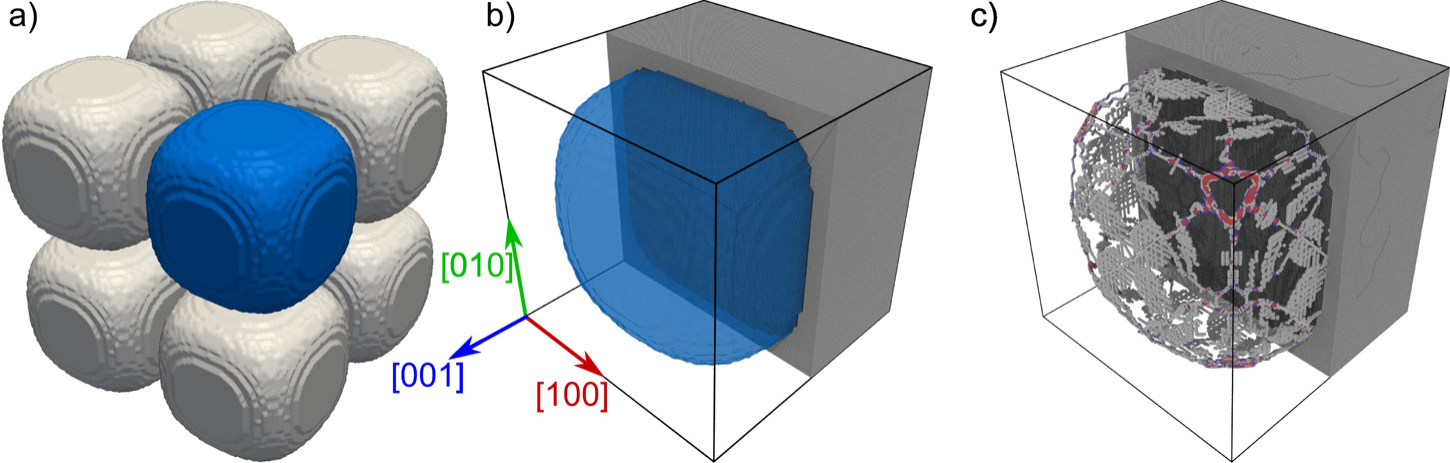
Generation of an atomistic sample using a snapshot of a phase field simulation: (a) periodic structure showing the surface topology of the *γ*′ particles as defined by the snapshot. Since the structure is periodic, only the *γ*′ particle marked in blue is used for the atomistic structure generation; (b) atomistic structure generated by *nano**sculpt* using different lattice constants for *γ* (*a*_0_ = 0.352 nm) and *γ*′ (*a*_0_ = 0.357 nm). The dimensions of the simulation box are 64 nm× 64 nm× 64 nm. Atoms are colored by the element type: grey–Ni, black—Al. The reconstructed *γ*′ particle is shown as a transparent blue surface. (c) Misfit dislocation network along the *γ*/*γ*′ interface (blue surface in b)), obtained as a result of energy minimization. Red atoms denote stacking faults, blue atoms denote atoms in bcc structure, and white atoms denote other defect atoms in the *γ* phase.

Fig. 4, Fig. 5 show two microstructures obtained by using snapshot structures of other simulation methods. The first one in Fig. 4 is a polycrytalline microstructure obtained from a snapshot of a vertex-dynamics grain-growth simulation [19], and comprises 107 grains. A salient feature of this structure is the presence of curved grain boundaries, while Voronoi tessellated structures invariably result in planar grain boundaries.

The second example in Fig. 5 is a *γ*/*γ*′ periodic microstructure of a Ni-base superalloy obtained from phase field simulations [20]. The size of the *γ*′ particle is 58 nm× 58 nm× 58 nm and is filled with Ni and Al atoms in a L1_2_ structure. The *γ* channels are of 8 nm width and are filled with Ni atoms in a *fcc* structure. The difference in lattice constants of the two phases results in a misfit dislocation network in the relaxed structure as shown in Fig. 5(c).

### Digitization of experimental micrographs

**Fig. 6:**
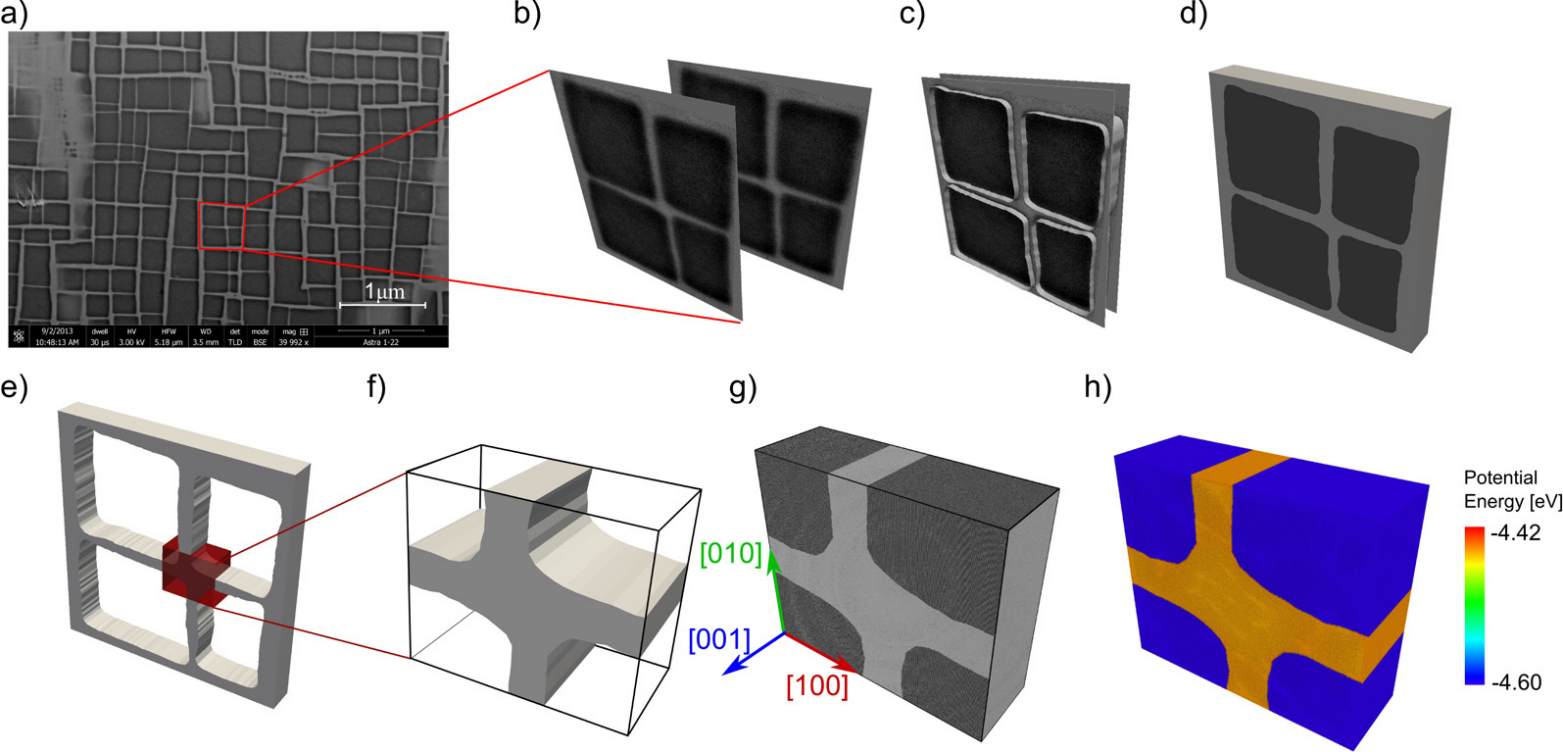
Generation of atomistic sample using experimental micrographs: (a) SEM micrograph of a Ni-base superalloy with *γ*/*γ*′ microstructure. Only the region of interest—demarcated by the red lines—consisting of four *γ*′ cubes is used for further processing. (b) The reduced micrograph is treated for greyscale values of missing pixels and duplicated to obtain front and back sections of the microstructure. (c) Using isolines of greyscale, we identify the interphase boundary and demarcate the *γ* channels and *γ*′ particles. (d) A surface mesh is now constructed individually around the different phases. (e) Only the surface mesh of the *γ* channel is retained from (d), since the *γ*′ phase is essentially a negative of the *γ* phase. (f) Since the size of individual particles is rather large (approx. 500 nm), only the mesh pertaining to the red cuboid box in (e) is extracted. By performing Boolean operations, an enclosed surface mesh is now obtained. (g) Atomistic structure with Ni in *γ* phase and Ni_3_Al in the *γ*′ phase. Ni atoms are colored gray, whilst Al atoms are colored black. (h) Potential energy in the relaxed structure of (g).

An elegant way of obtaining realistic structures for atomistic simulations is to directly use experimental data. One approach is to digitize micrographs of SEM or TEM investigations. We demonstrate the methodology using a SEM micrograph of a Ni-base superalloy [21], see Fig. 6(a). We first identify a region of interest, e.g., comprising of four *γ*′ particles (Fig. 6(b)). This region must be sufficiently large so as to identify the different phases and construct a surface mesh that encompasses individual phases. The different phases are now demarcated using isosurfaces of greyscale or colors in the picture, as shown in Fig. 6(c). Surface meshes are then individually constructed over each distinct phase (Fig. 6(d)). We then proceed to fill the individual phases with atoms. Since the size of the *γ*′ particles is very large (approximately 500 nm in length), for the current example we cut out a portion that includes the intersection of horizontal and vertical channels, see Fig. 6(e,f). The *γ*′ particles are then filled with Ni and Al atoms in a L1_2_ structure, while the *γ* channels are filled with Ni atoms in a *fcc* structure. A characteristic orientation of 〈100〉 ∥ *x* axis along with an average lattice constant of 0.3545 nm as defined by the interatomic potential of Mishin [22] is used for both phases whilst generating the structure (Fig. 6(g)). A drawback of this method is that it is generally limited to 2D or quasi 2D structures as seen in the current example. However, using image stacks obtained by serial sectioning [23] a fully 3D structure can be obtained. The downside, however, is that individual images have to be aligned perfectly so as to obtain accurate topological information in the third dimension. An alternate method is to use artificial methods like the Voronoi tessellation in the third dimension (e.g. [24]) to obtain a full 3D microstructure.

### Three-dimensional tomography

Tomographic techniques like X-ray tomography [25], electron tomography [26] and atom probe tomography [2] are being increasingly used in materials research. They have garnered significant interest since they facilitate complete 3D—and sometimes even 4D (spatial and temporal)—characterization of the microstructure [27]. Atom probe tomography, in particular, provides information on the chemical composition of the microstructure with atomic-level resolution and a relatively large field-of-view. The technique combines time-of-flight mass spectroscopy with a position-sensitive detector to estimate the coordinates of individual ions that are sequentially evaporated from a needle-shaped specimen. Although the positional and chemical information of individual atoms is ideal input data for atomistic simulations, the partial loss of evaporated ions (current detection rate in APT is 35–80%) and field evaporation artefacts affecting the accuracy of reconstructed atomic positions hinder the direct usage of APT datasets in atomistic simulations and, in general, require lattice rectification/reconstruction algorithms [28] for the reconstruction of the APT sample [29].

Recently, Prakash et al. [1] proposed an alternate way to APT-informed atomistic simulation samples. The fundamental idea is to exploit the chemical information obtained from APT in order to construct iso-density surfaces that demarcate the boundary between different regions in the specimen. The key here is to use an ion that segregates strongly into the region of interest. The methodology of obtaining an APT-informed atomistic specimen is demonstrated on a dataset of a Ni-base superalloy LEK94.

**Fig. 7:**
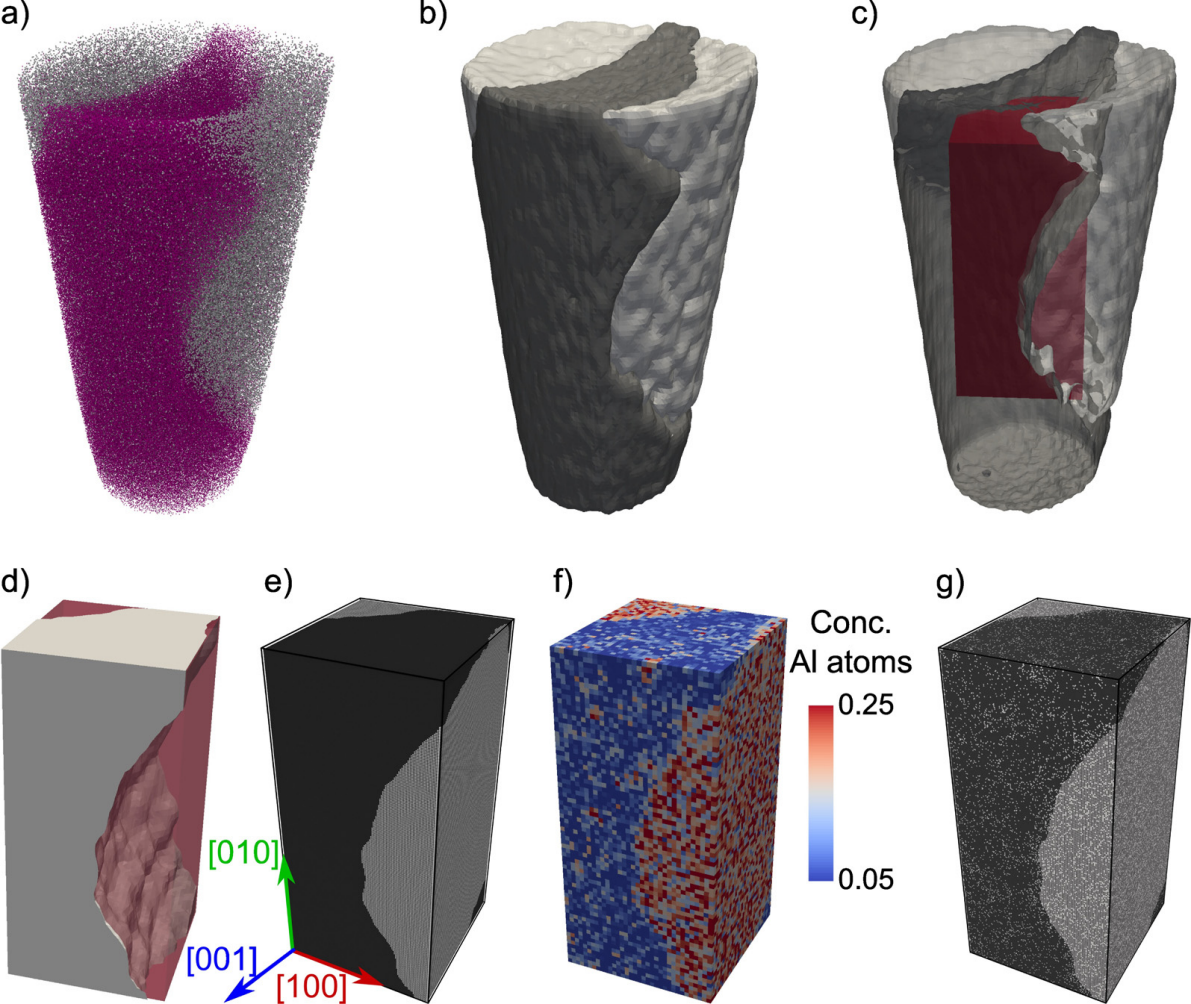
Generation of the APT-informed atomistic sample: (a) APT sample with only atoms identified as Ti (gray) or Re (magenta) displayed; (b) iso-density surfaces of Re defining the *γ*-channel (black) and Ti defining the *γ*′ particles (gray) of sample in (a); (c) simulation box (marked in red) that is to be cut out of the isosurface definition. For clarity the isosurfaces are displayed as semi-transparent; (d) simulation box with the isosurface identifying the *γ* phase. The *γ*′ phase is generated as the negative of the *γ* phase; (e) stoichiometric atomistic sample with Ni (fcc) in the *γ* phase and Ni_3_Al (L1_2_) in the *γ*′ phase; (f) concentration of aluminum ions in the original APT sample; (g) non-stoichiometric atomistic sample generated by stochastically replacing atoms so as to obtain local atomic concentrations like those seen in (f).

The first step in the construction of an APT-informed simulation sample is to identify the interface between different phases. This can be done by using the position density of specific ions that partition strongly into individual phases. In the example of the *γ*/*γ*′ microstructure shown below, we identify the interphase boundary based on the density of Re and Ti ions, since they are mostly to be found in the *γ*-channel and *γ*′ particles, respectively (Fig. 7(a)). As an alternative, one could also use Al ions since they are to be mostly found in the *γ*′ particle.

We then construct an isosurface that encompasses the different phases. In the current example, the isosurfaces are constructed by means of a marching cubes algorithm [30] implemented in the R statistical software [31]. Fig. 7(b) shows the iso-density surfaces of Re and Ti ions obtained for position density values of 85% and 75%, respectively. Both isosurfaces were obtained independent of all other ions in the sample. The iso-density values were chosen so as to obtain complementary definition of the interface; with the aforementioned values an almost identical definition of the interface is obtained, as seen in Fig. 7(b,c).

Following the generation of the isosurfaces, we cut out a cuboidal box that represents the desired simulation sample (Fig. 7(c,d)). Care is taken to avoid boundary artefacts (like extremely small interfaces) whilst preserving topologically relevant features of the APT sample. The atomistic sample is then generated using *nano**sculpt* in two stages, identical to the procedure followed for the SEM-image informed sample. In a first stage only the region pertaining to *γ*-channel is filled with Ni atoms in a *fcc* structure, and in a second stage, the regions corresponding to *γ*′ particles are filled with Ni_3_Al in a L1_2_ structure. During this generation of the APT-informed atomistic sample, only the iso-density surface of Re is used in order to have a unique definition of the interface. The lattice orientation corresponding to that of the original single crystal from which the APT sample was extracted, and an average lattice constant of 0.345 nm—as specified by the interatomic potential of Mishin [22]—was used for both the *γ* and *γ*′ phases. The generated atomistic structure is shown in Fig. 7(e) and contains approximately 16 million atoms.

The advantage of APT over other tomography techniques is the availability of chemical mapping, i.e. information on the chemical species of different ions. Consequently, one can incorporate not just structural features in atomistic samples as shown above, but also concentrations of different chemical species (see Fig. 7(f)). This can be accomplished by stochastically replacing atoms in the stoichiometric simulation sample, i.e. Ni and Al, so as to conform to local concentrations of the corresponding atoms in the original APT sample (see Fig. 7(g)). Such a non-stoichiometric sample generated from concentration data of Ni and Al obtained by a voxelized approach is shown in Fig. 7(g). Further refinement can be obtained through Monte Carlo simulations, following e.g. [29], in order to incorporate short range effects.

## Additional information

### Remarks on the use of isolines and/or isosurfaces

We note that the usage of isolines and isosurfaces in the construction of the atomistic samples has an inherent ambiguity associated with it. The topology of the interface can depend significantly on the choice of the isolevel. Consequently, the constructed interface needs to be validated and verified by alternate means. This can be done in a subjective manner, i.e. by verifying if the reconstructed isosurface is approximately at the same position as can be perceived in the original dataset. In the example of the SEM micrograph informed sample above, the constructed isosurface seemingly forms the interface between the *γ* and *γ*′ phases. Changing the isolevel of the greyscale changes the size of the particle but does not lead to a drastic change in the topology of the interface. A more robust and objective method is shown in the example of the APT informed sample; using information of two different ions that partition strongly into either the *γ* or the *γ*′ phases, the interface defined by the iso-density surface of one ion can be verified by the interface predicted by the second ion.

### Software details

The software *nano**sculpt* is available as an *open-source* tool [32] and is licensed under the GNU General Public License (GPL) v3. It is a simple command-line driven tool that is programmed to work on Linux-based operating systems, and has been successfully tested on the openSUSE operating system.

The requisite input for *nano**sculpt* includes the surface mesh of the enclosed volume and crystallographic information (orientation, lattice constant and mass of individual species of atoms). The program then reads in this data (provided through a parameter file) and generates an atomistic structure that fills the given enclosed volume. It is the responsibility of the end user to provide all requisite input in the right format. Currently, the input surface mesh needs to be provided in the Wavefront OBJ format [33], which is a universally accepted open format for 3D graphics applications. The generated output is in a format suitable for simulations with the open-source massively parallel molecular dynamics code IMD [34]. Nonetheless, due to its modularized structure, *nano**sculpt* is easily extendable to include other input and output formats.

There are no specific hardware dependencies for generating structures with *nano**sculpt*. Nonetheless, we recommend using a system with substantial amount of memory to ensure speedy computations. Ballpark computation times were found to be approximately 1 cpu hour for 150 million atoms. As an example, the rack and pinion system in Fig. 3, with 130 million and 570 million atoms, respectively, takes roughly 3.4 cpu hours on a system with Intel Xeon E5 processor (2.3 GHz) and 128 GB of RAM.

## Conclusions

In this work, we have presented a methodology for generating realistic samples for atomistic simulations. With this methodology, topologically relevant features like curvature, neighborhood information in polycrystalline samples, etc. can be easily incorporated into studies of qualitative nature that focus on e.g., defect–defect interaction mechanisms.
